# Determinants of pre-hospital pharmacological intervention and its association with outcome in acute myocardial infarction

**DOI:** 10.1186/s13049-015-0188-x

**Published:** 2015-12-01

**Authors:** Rasmus Strandmark, Johan Herlitz, Christer Axelsson, Andreas Claesson, Anders Bremer, Thomas Karlsson, Maria Jimenez-Herrera, Annica Ravn-Fischer

**Affiliations:** Department of Metabolism and Cardiovascular Research, Institute of Internal Medicine, Sahlgrenska University Hospital, Johan Herlitz office, Registercentrum i Västra Götaland, 413 45 Gothenburg, Sweden; Department of Metabolism and Cardiovascular Research, Institute of Internal Medicine, Sahlgrenska University Hospital, Gothenburg, Sweden; The Prehospital Research Centre Western Sweden, University of Borås, Borås, Sweden; Department of Medicine, Center for Resuscitation Science, Karolinska Institute, Stockholm, Sweden; Centre for Applied Biostatistics, Occupational and Environmental Medicine, Sahlgrenska Academy at University of Gothenburg, Gothenburg, Sweden; Department of Nursing, Universitat Rovira i Virgili, Tarragona, Spain; Department of Cardiology, Sahlgrenska University Hospital, Gothenburg, Sweden

**Keywords:** Chest pain, Myocardial infarction, Emergency medical services, Early medical intervention, Aspirin, Nitroglycerin

## Abstract

**Background:**

The aim of this study was a) To identify predictors of the use of aspirin in the pre-hospital setting in acute myocardial infarction (AMI) and b) To analyze whether the use of any of the recommended medications was associated with outcome.

**Methods:**

All patients with a final diagnosis of AMI, transported by the Emergency Medical Services (EMS) and admitted to the coronary care unit at Sahlgrenska University Hospital in Gothenburg, Sweden, in 2009–2011, were included.

**Results:**

1,726 patients were included. 58 % received aspirin by the EMS. Ischemic heart disease (IHD) was suspected in 84 %. Among patients who did not receive aspirin IHD was still suspected in 67 %. Among patients in whom IHD was suspected, and who were not on chronic treatment with aspirin the following predicted its pre-hospital use: a) age (odds ratio 0.98; 95 % confidence interval (CI) 0.96–0.99); b) a history of myocardial infarction (2.21; 1.21–4.04); c) priority given by EMS (8.07; 5.42–12.02); d) ST-elevation on ECG on admission to hospital (2.22; 1.50–3.29); e) oxygen saturation > 90 % (3.37; 1.81–6.27).

After adjusting for confounders among patients who were not on chronic aspirin, only nitroglycerin of the recommended medications was associated with a reduced risk of death within 1 year (hazard ratio 0.40; 95 % CI 0.23–0.70).

**Conclusions:**

Less than six out of ten patients with AMI received pre-hospital aspirin. Five clinical factors were independently associated with the pre-hospital administration of aspirin. This suggests that the decision to treat is multifactorial, and it highlights the lack of accurate diagnostic tools in the pre-hospital environment.

Nitroglycerin was independently associated with a reduced risk of death, suggesting that we select the use for a low-risk cohort.

**Electronic supplementary material:**

The online version of this article (doi:10.1186/s13049-015-0188-x) contains supplementary material, which is available to authorized users.

## Background

With the exception of fibrinolysis [1], the evidence for the use of medication before hospital admission in suspected acute myocardial infarction (AMI) is limited [2]. Despite this fact, pre-hospital pharmacological intervention has been routine in most developed countries for at least 25 years. The drugs recommended by guidelines have remained largely unchanged and include oxygen, aspirin, nitroglycerin and morphine. In addition, other drugs, such as clopidogrel and beta-blockers, have been introduced as pre-hospital treatment but then often on a more strict indication.

Most studies of pharmacological interventions in AMI have been performed in hospital settings. Even though interventions with oxygen, nitroglycerin, aspirin and morphine have been widely accepted and used for a long time, the scientific evidence of their benefit is limited and in some cases contradictory. A Cochrane report concluded that the underlying evidence for the widespread use of oxygen in AMI is suggestive of possible harm [3]. The use of morphine in AMI has been shown to relieve pain [4, 5], but it has not been shown to improve outcome. On the contrary, in cases of unstable angina pectoris and non-ST-elevation myocardial infarction, morphine has been associated with an increased mortality rate [6].

Nitroglycerin has been extensively studied in acute coronary syndrome (ACS). The ISIS-4 and GISSI-3 trials convincingly demonstrated that the continued treatment with nitroglycerin after a coronary event had no prognostic benefit [7, 8]. On the other hand, a recent Cochrane report concluded that, if used within the first 24 h, nitroglycerin is associated with a reduced risk of death within the first two days [9]. This suggests that nitroglycerin is of the greatest benefit early in the treatment of AMI. The risks and potential benefit of very early treatment with nitroglycerin in the pre-hospital setting have, however, only been sparsely studied. To our knowledge, no randomized, controlled trial has been conducted on the subject. In observational studies, the pre-hospital administration of sublingual nitroglycerin is reported to be safe, with the predominant adverse effect being hypotension (with an incidence of 0.7–3.2 %) [10, 11], and to be associated with reduced chest pain [10].

Aspirin has been shown to have a positive effect on outcome in AMI. In 1988, the ISIS-2 incontrovertibly established aspirin as an integral part of the treatment in AMI [12]. Although ISIS-2 did not clearly document that very early treatment is better than later administration of aspirin (e.g. within the first 24 h), it soon became routine in many countries, including Sweden, to administer aspirin pre-hospitally. The current guidelines state that an oral loading dose of 300 mg should be given as early as possible by the EMS on suspicion of ACS [13]. The pre-hospital administration of aspirin has been reported to be safe [14], but the true benefit of this strategy has never been documented. Observational studies comparing the outcome among patients who received very early aspirin with those who received it later on have produced conflicting results [15–18]. Furthermore, the adherence of EMS clinicians to the recommendations has been reported to vary, with pre-hospital aspirin being administered to ideal candidates in 33–62 % of all cases [19–22]. One small study exploring the reasons for this relatively low adherence concluded that the leading reason for the EMS provider not to administer aspirin was that the chest pain was not believed to be of cardiac nature [21].

Aspirin has proven benefits, with a tendency towards greater benefit with very early treatment [12]. This possibly makes it the most important intervention in the pre-hospital setting. We therefore aimed to identify predictors of the use of aspirin in the pre-hospital setting in AMI and to analyze whether the use of any of the recommended medications was associated with outcome. A secondary objective was to assess predictors for a suspicion of ischemic heart disease (IHD) by the EMS clinician.

## Methods

### Study settings

The county of Västra Götaland is the second largest county in Sweden, with approximately 1.6 million people in an area of 24,000 km^2^. Sahlgrenska University Hospital in Gothenburg (600,000 inhabitants) is one of four percutaneous coronary intervention (PCI) centers in the county.

In Sweden, 112 is the common public emergency phone number for all emergency services. At the time of the study, all calls to the dispatch center on medical issues were redirected to a nurse, whom through a systemized caller-interrogation, using decision support software, determined a response output category for the case: (1) Immediately life threatening; (2) Urgent, but not life threatening; (3) A reasonable waiting time is not considered to influence the patients condition.

The ambulances were staffed by one nurse clinician, responsible for the pre-hospital triage and care, and one emergency medical technician. The educational level of nurse clinicians in the EMS was three years university studies to achieve a degree of Bachelor in nursing, and one additional year to achieve a degree of Master in pre-hospital nursing. A pre-hospital triage protocol (METTS-Pre) and local EMS guidelines were used as decision support. All the ambulances were equipped with a monitor/defibrillator. The pre-hospital ECGs were assessed manually. If ECG abnormalities were detected, the ECG was sent, using telemedicine, to the corresponding coronary care unit (CCU) for evaluation by a nurse at the CCU and, if necessary, with the support of an on-call cardiologist. If ST-elevation or a left bundle branch block (LBBB) were present, the patient was transported to the CCU or directly to the catheterization laboratory, bypassing the emergency department (ED). If not, the patient was transported to the nearest ED. After assessment by an ED physician, a large proportion was subsequently transferred to the CCU on suspicion of AMI (and thus included in this study).

The decision to administer oxygen, sublingual nitrates, intravenous morphine and oral aspirin was at the discretion of the nurse clinician. The standard dose of aspirin was 300 mg. After telephone contact with the receiving cardiologist, intravenous beta-blockade (rarely) and/or oral clopidogrel could also be administered pre-hospitally.

### Population and data collection

This study comprised consecutive patients admitted to the CCU at Sahlgrenska University Hospital between 1 January 2009 and 31 December 2011, with a final diagnosis of AMI (ICD-10: I21-I22) at discharge. Thus, all types of AMI were included in the analyses. All the patients had been transported to hospital by the EMS. Data were obtained from the Register of Information and Knowledge about Swedish Heart Intensive care Admissions (RIKS-HIA). The list consisted of 2,524 events. The matching EMS casebook was identified in the medical log program used by EMS in the county of Västra Götaland; AmbuLink. In total 798 cases were excluded before analysis. The reasons for exclusion were: a) the transport was made by EMS from another county or the relevant EMS casebook was missing (*n* = 280), b) the transport was from a referral hospital, where diagnosis and initial treatment already had been given (any prior transport to the ED at the referral hospital was however included) (*n* = 164), c) the transport was from a local health center, where treatment already had been initiated (*n* = 252), d) cases involving a cardiac arrest, drastically altering treatment strategy (*n* = 97) and e) missing information on pre-hospital aspirin (*n* = 5).

In the excluded cases it was not regarded as meaningful to evaluate the use of aspirin since such treatment had already been started by others.

### Statistical methods

Data are presented as crude (i.e. not age-adjusted) rates. All *p*-values in Tables 1 and 2 and Additional file 1: Tables S1 and S2, except for age itself, are age adjusted. Age was compared using the Mann–Whitney U-test. Logistic regression was used to calculate age-adjusted *p*-values for difference in proportions. To identify predictors of the pre-hospital use of aspirin among patients with suspected IHD and not on previous chronic aspirin, multiple logistic regression was used, in a forward stepwise selection mode, with *p* < 0.01 as a criterion for staying in the model. Variables tested for inclusion in the model were those relevant baseline factors with a univariate p-value below 0.05 for association with pre-hospital aspirin in this group of patients. The same strategy was used to identify predictors of IHD assessment in all patients. See Additional file 1 for a complete list of variables tested.

**Table 1:** Medication administered by EMS (%)

Pre-hospital aspirin				
	All (*n* = 1726)	Yes (*n* = 995)	No (*n* = 731)	p*
Oxygen	78	88	62	<0.0001
Nitroglycerine	66	82	45	<0.0001
Aspirin	58	100	0	-----
Morphine	57	76	30	<0.0001
Beta-blockers	2	2	1	
Clopidogrel	38	64	3	<0.0001

*Age adjusted *p*-value, denoted if <0.05

**Table 2:** Age, gender and previous history (%, if not otherwise stated)

Prehospital aspirin				
	All (*n* = 1726)	Yes (*n* = 995)	No (*n* = 731)	p*
Age (mean ± SD)	70 ± 13	68 ± 13	73 ± 13	<0.0001
Women	33	29	39	0.03
Previous history				
Diabetes (7)^a^	20	18	23	0.01
Hypertension (21)^a^	44	42	47	
Smoking (98)^a^	27	30	24	
Myocardial infarction (19)^a^	28	22	36	<0.0001
Heart failure (72)^a^	9	5	13	<0.0001
PCI (25)^a^	16	13	19	0.0008
Heart surgery (14)^a^	9	7	12	0.001
Stroke (19)^a^	10	7	14	<0.0001
Medication				
Aspirin (27)^a^	38	31	46	<0.0001
Any other cardiovascular drug (51)^a^	61	54	71	<0.0001

*Age adjusted *p*-value (except for age itself), denoted if <0.05

^a^Number of patients with missing information

For mortality analyses, Kaplan-Meier estimates were used and age-adjusted *p*-values and hazard ratios with corresponding confidence intervals were calculated using Cox’s proportional hazards model. The association between the six recommended medications and one-year mortality among patients with no previous aspirin was analyzed for each medication separately and adjusted for all baseline and in-hospital treatment variables with a univariate *p* < 0.10 for association with mortality (see Additional file 1). All the tests were two sided and *p*-values below 0.01 were considered statistically significant. All the analyses were performed using SAS for Windows version 9.3.

### Ethical considerations

This study has been conducted within the framework of a master’s thesis at Sahlgrenska University Hospital and is therefore not subjected to review by the Swedish Ethical Reviewer Boards. However, the work was done according to the Swedish law (SFS) 2003:460; the law of ethical considerations and human trials, and according to the Helsinki Declaration. In all the analyses, patients remained anonymous and patient integrity was thus respected. Patients included in RIKS-HIA have been given information about participation in the register and have been given the opportunity to decline.

## Results

### Medication administered by EMS

In all, 1,726 patients were transported by the EMS to hospital and fulfilled the criteria for AMI. Table 1 shows the use of various medications. Pre-hospital aspirin was administered in 58 % of the cases. The administration of oxygen, nitroglycerin, morphine and clopidogrel was more common in the group receiving aspirin.

### Age, gender and previous history

The overall mean age was 70 years, and 33 % were women. Patients that received aspirin were younger than those that did not, and had fewer indications of previous cardiovascular disease, as shown in both their previous history and their previous chronic medication (Table 2).

### Symptoms and initial assessment by dispatchers and EMS

In 92 % of all cases a pre-hospital ECG was recorded and the nurse clinician suspected IHD as the underlying etiology in 84 % of all cases. Among patients who received aspirin there was an initial suspicion of IHD in 96 %, whereas among patients who did not receive aspirin a suspicion of IHD was raised in only 67 % (*p* < 0.0001). When comparing patients who received aspirin with those who did not there were many differences in terms of symptoms, initial assessment and hemodynamics. In summary, patients who received aspirin differed from those who did not in that they were given a higher priority by the dispatchers and by the EMS; chest pain and pain in the arms were more prevalent; they more often had cold sweat and nausea; and they had less tachycardia and less oxygen desaturation. For details see Additional file 1: Table S1.

### Status on admission to hospital, treatment and investigation in hospital and 1 year mortality

Fifty-eight percent of the patients had ST-elevation on first in-hospital ECG. ST-elevation was more common among the patients that had received pre-hospital aspirin. When comparing patients who received aspirin with those who did not there were differences in the use of various medications after hospital admission. Investigations such as coronary angiography and echocardiography were performed more frequently among those who received aspirin. For more details see Additional file 1: Table S2.

The overall one-year mortality was 13.6 %. Patients who were given aspirin pre-hospitally had a lower one-year mortality than those who did not (10.1 % versus 18.6 %; *p* = 0.009).

### Predictors of the administration of aspirin by EMS

In this analysis we only included patients in whom there was a suspicion of IHD and excluded patients on chronic medication with aspirin. Five independent predictors of the use of aspirin were identified, as shown in Table 3.

**Table 3:** Predictors of the use of aspirin prior to hospital admission among patients with suspected IHD and not on previous chronic aspirin medication

	OR^a^	(95 % CI)^a^	*p*
Age (per year)	0.977	(0.963, 0.991)	0.001
Previous history			
Myocardial infarction	2.21	(1.21, 4.04)	0.01
EMS assessment			
Priority	8.07	(5.42, 12.02)	<0.0001
ECG pattern			
ST-elevation	2.22	(1.50, 3.29)	<0.0001
Cardio-respiratory finding			
Oxygen saturation ≥90 %	3.37	(1.81, 6.27)	0.0001

^a^Odds ratio (95 % confidence interval)

### Pre-hospital pharmacological intervention in relation to one-year mortality

When adjusted for all potential confounders recorded, the only drug associated with a reduced risk of death within one year of follow-up was nitroglycerin (Table 4).

**Table 4:** Prehospital pharmacological intervention in relation to 1 year mortality among those not on previous chronic aspirin medication (*n* = 1061) – each intervention analyzed separately

	HR^a^	(95 % CI)^a^	*p*
Adjusted for confounders# from Tables 2, 3 and 4			
Oxygen	0.97	(0.48, 1.95)	0.92
Nitroglycerine	0.40	(0.23, 0.70)	0.001
Aspirin	1.14	(0.60, 2.16)	0.69
Morphine	1.13	(0.62, 2.06)	0.69
Beta-blockers	1.45	(0.18, 11.76)	0.73
Clopidogrel	1.06	(0.56, 1.99)	0.86

^a^Hazard ratio (95 % confidence interval)

^#^All factors with age adjusted *p* < 0.10 for association with 1 year mortality

### Predictors of assessment as ischemic heart disease

There were seven predictors for such an assessment by the EMS clinician (Table 5).

**Table 5:** Predictors of assessment as ischemic heart disease

	OR^a^	(95 % CI)^a^	*p*
Symptoms			
Chest pain	15.67	(10.27, 23.90)	<0.0001
Pain in arms	2.45	(1.58, 3.80)	<0.0001
Cold sweat	1.75	(1.15, 2.65)	0.009
Dispatch center assessment			
Priority	1.70	(1.20, 2.41)	0.003
ECG pattern			
ST-elevation	2.67	(1.83, 3.89)	<0.0001
No pathologic T-wave	2.95	(1.47, 5.91)	0.002
Cardio-respiratory finding			
Oxygen saturation ≥90 %	2.16	(1.36, 3.42)	0.001

^a^Odds ratio (95 % confidence interval)

## Discussion

The three major results from this study are as follows:

- Fifty-eight percent of the patients with a final diagnosis of AMI transported by the EMS in the Municipality of Gothenburg received aspirin by EMS before hospital arrival.
- A variety of clinical factors were associated with the pre-hospital use of aspirin by EMS. They reflect the patients’ age, previous history, the initial assessment made by the EMS clinicians, the initial ECG pattern and the patients’ cardiorespiratory status (oxygen saturation).
- The pre-hospital use of nitroglycerin, when adjusted for confounders, was associated with a reduced risk of death during one year of follow-up.

### Use of aspirin

In previous reports from Sweden and other countries, the proportion of patients with suspected AMI treated with aspirin pre-hospitally varies between 33 % and 62 % [15, 19–22] and the 58 % in the present study is thus in line with previous findings. There are several possible explanations as to why adherence to guidelines was so low. In an earlier study, the main reason for EMS providers not to administer aspirin was that the chest pain was not believed to be of cardiac nature [21]. In the present study the nurse clinician stated IHD as the suspected etiology in 84 % of all cases, indicating that in some cases the nurse clinician failed to identify the condition as an ACS. In these cases, the decision not to treat is the logical consequence of the assessment. However, among the patients not receiving aspirin the nurse clinician still suspected IHD in 67 % of the cases. The reasons for withholding treatment in these cases are not apparent. One might be that a contraindication was present, although the only contraindication stated in local EMS guidelines at the time was known allergy to acetylsalicylic acid. Emesis might be a more common issue hindering the administration of aspirin (in Sweden, aspirin is only available in oral dosage forms). Another practical issue to consider is the time available for interventions. In an urban setting, the hospital is sometimes only minutes away and the nurse clinician might have to prioritize other actions.

Recent self-administration of aspirin was the second most prevalent reason EMS providers gave for not administering aspirin, in the previous study [21]. In this study 38 % of the patients were prescribed aspirin for regular use prior to the event. This might have influenced the nurse clinicians decision, even though guidelines did not state that the loading-dose of aspirin should be withheld from these patients.

### Predictors of the use of aspirin

A history of myocardial infarction, and an initial suspicion by the EMS clinician that IHD was the underlying etiology, was associated with a higher use of aspirin, indicating adherence to guidelines. High priority given by the EMS clinician was a strong predictor of the use of aspirin. In Gothenburg at the time, all pre-hospital ECGs with an ST-elevation or a LBBB (but also other abnormalities) were sent to the CCU at Sahlgrenska University Hospital. A nurse and/or an on-call cardiologist decided whether the patient was to be accepted for direct transport to the cardiac catheterization laboratory, the CCU, or referred to the nearest ED. The patients accepted for direct transport were often given high priority and this was in turn a strong predictor of aspirin treatment.

On the other hand, patients referred to the nearest ED received lower priority and therefore most probably had a longer mean transportation time as a group. This should have given the nurse clinician more time to administer treatment, but this group was instead less likely to receive aspirin. This clearly reflects the possibility that a referral to the nearest ED was interpreted by the nurse clinician as an “acquittal”, and that further interventions were therefore unnecessary. If this is indeed a prevalent interpretation, it reflects the importance of continuing education. In the future, the CCU nurse or the on-call cardiologist might also give advice about early treatment in this subset.

The observation that a ST-elevation on the hospital admission ECG, also predicted a higher use of aspirin is in agreement with previous statements. In these cases, the ambulance was redirected to the catheterization laboratory or to the CCU, and the on-call cardiologist often prescribed oral clopidogrel to be administered pre-hospitally, in addition to standard treatment, making the decision not to administer aspirin at the same time controversial.

Oxygen saturation of ≥ 90 % predicted an increase in the use of aspirin. This is most probably explained by a more stable clinical condition in these patients. Patients with low oxygen saturation are often in a bad clinical condition creating problems with oral medication.

A lower age was associated with the use of aspirin suggesting that age itself is a predictor of adherence to guidelines.

The finding that patients who received aspirin in the pre-hospital setting were more often revascularized is most likely an independent finding, simply reflecting the high incidence of ST-elevation AMI among these patients.

The proportion of patients treated with coronary artery bypass grafting was very low and in agreement with previous findings [23].

### Predictors of death

The use of nitrates was associated with a reduced risk of death after adjustment for confounders. Nitrates have been widely used in ACS for many years, are well tolerated [10, 11] and are associated with a reduction in chest pain in the pre-hospital setting [10]. To our knowledge, no randomized placebo-controlled pre-hospital trial, investigating the effects of nitrates, has been performed. Two large, randomized, placebo-controlled trials with randomization after hospitalization have not shown any benefit in terms of outcome [7, 8]. One explanation of our findings is that there are confounders that were not adjusted for. It is likely that patients with severe pain, often those with ST-elevation, and without hemodynamic complications, were more frequently given nitroglycerin. These patients are reported to have a good outcome [24]. However, we adjusted for ECG findings and for hemodynamic complications. Another, perhaps less likely, explanation is that nitroglycerin, when given in the very early phase of acute myocardial ischemia, has a beneficial effect on the myocardium. A recent Cochrane report that included 18 trials of nitrates in patients with an acute cardiovascular event concluded that nitrates are associated with increased survival if administered within the first 24 h [9].

### Predictors of assessment as ischemic heart disease

IHD was suspected in 84 % of all cases. The predictors of such a suspicion included typical symptoms, a high priority by the dispatcher, typical ECG signs and a normal oxygen saturation. These findings suggest that the EMS clinicians, to a great extent, had the knowledge and the ability to detect AMI in patients with classical symptoms and signs. However, far from all cases of AMI presents itself with this symptom complex. Our findings suggest that the EMS clinicians’ ability to detect AMI in patients with atypical symptoms is limited. Today, pre-hospital decision-making is based on clinical history and clinical findings. However, recent data suggest that the use of biochemical markers in the pre-hospital setting might help to further improve the diagnostic accuracy [25].

### Limitations

This is an observational, retrospective study. The study was performed in an urban area with relatively short transport times. The results might have been different, had the study been undertaken in a rural area, even though patients transported from other areas of the county were frequent and not excluded.

The patients were recruited from the RIKS-HIA register. At the time this register covered about ninety percent of all patients hospitalized with AMI under the age of 80 years. Older patients with severe co-morbidity are not always admitted to the CCU and are therefore not included in the register [23]. This, in combination with the fact that this study only included patients transported with the EMS, may explain the high figure of ST-elevation (58 %). In turn, this might explain why the overall one-year mortality was relatively low (14 %).

We assume that the total number of patients admitted to Sahlgrenska University Hospital, and having a final diagnosis of AMI, was about 4000 during the inclusion period. This figure is based on previous surveys [23]. The relatively large proportion of patients with ST-elevation in our survey suggests that the pre-hospital aspirin-use might have been even lower, had all patients with AMI been included.

Two clinical parameters (ECG findings and signs of heart failure) were recorded on admission to hospital instead of pre-hospitally, due to difficulties retrieving this information from the EMS chart. If information about the pre-hospital ECG had been available, it is possible that the proportion of patients with a pathological finding would have been different, as both time, and pre-hospital medication might have influenced the ECG pattern.

Patients who had taken aspirin (typically 75 mg) before EMS arrival constitute a confounding factor, when relating aspirin use to outcome. These patients were analyzed as though they did not receive aspirin and were therefore not included in the multivariate analyses.

## Conclusion

Less than six out of ten patients with AMI received pre-hospital aspirin. Five clinical factors were independently associated with the pre-hospital administration of aspirin. This suggests that the decision to treat is multifactorial, and it highlights the lack of accurate diagnostic tools in the pre-hospital environment.

Nitroglycerin was independently associated with a reduced risk of death, suggesting that we select the use for a low-risk cohort.

### Clinical implications

The lack of scientific evidence for the use of various medications prior to hospital admission is a clinical dilemma. Is the current strategy of administering several oral and intravenous drugs pre-hospitally, thereby possibly delaying transport, of the greatest benefit to the patient? This needs to be evaluated in future research.

Less than six out of ten patients with an AMI received aspirin by the EMS. Since the lack of suspicion of IHD and low priority were linked to a lower use of aspirin, we need better instruments to detect AMI in the early phase. We also need clarified EMS guidelines regarding aspirin administration to patients already on chronic treatment.

Furthermore, our data suggest that we select the use of nitroglycerin for a low-risk cohort. Any possible beneficial effect of nitroglycerin needs to be highlighted in a randomized trial.

## Additional file

Additional file 1:
**List of baseline variables and additional tables. ** Baseline variables tested for inclusion in the model identifying predictors of the use of aspirin, prior to hospital admission, in patients with no previous chronic aspirin medication and with a suspicion of IHD. Baseline variables tested for inclusion in the model identifying predictors of assessment as ischemic heart disease, in all patients. Baseline and in-hospital treatment variables used for adjustment when analyzing association between each of the six recommended pre-hospital medications and one-year mortality, in patients with no previous chronic aspirin medication. **Table S1**, displaying symptoms and initial assessment by dispatchers and EMS. **Table S2**, displaying status on admission to hospital, treatment and investigation in hospital, 30 days and 1 year mortality. (DOC 35 kb)
