# Supplementary material for: Determinants of pre-hospital pharmacological intervention and its association with outcome in acute myocardial infarction
Source: Scand J Trauma Resusc Emerg Med. 2015 Dec 1;23:105. doi: 10.1186/s13049-015-0188-x (PMC4665872; doi:10.1186/s13049-015-0188-x)
Supplement: Additional file 1: — List of baseline variables and additional tables. Baseline variables tested for inclusion in the model identifying predictors of the use of aspirin, prior to hospital admission, in patients with no previous chronic aspirin medication and with a suspicion of IHD. Baseline variables tested for inclusion in the model identifying predictors of assessment as ischemic heart disease, in all patients. Baseline and in-hospital treatment variables used for adjustment when analyzing association between each of the six recommended pre-hospital medications and one-year mortality, in patients with no previous chronic aspirin medication. Table S1, displaying symptoms and initial assessment by dispatchers and EMS. Table S2, displaying status on admission to hospital, treatment and investigation in hospital, 30 days and 1 year mortality. (DOC 35 kb) [file 13049_2015_188_MOESM1_ESM.doc]

**Supplement**

Baseline variables tested for inclusion in the model identifying predictors of the use of aspirin, prior to hospital admission (i.e. those with an univariate p<0.05 for association with the use pre-hospital aspirin), in patients with no previous chronic aspirin medication and with a suspicion of IHD:

Age

Previous myocardial infarction

Previous heart failure

Previous PCI

Previous heart surgery

Any previous chronic cardiovascular medication (other than aspirin)

Priority at the dispatch center

Priority by EMS

Pain/discomfort in neck

Pain/discomfort in arms

Dyspnea symptoms

Cold sweat symptoms

Nausea symptoms

Paleness symptoms

Heart rate >100 beats/min

Oxygen saturation <90%

ST-elevation on first in-hospital ECG

ST-depression on first in-hospital ECG

Pathologic T-wave on first in-hospital ECG

Pulmonary rales on admission to hospital

Baseline variables tested for inclusion in the model identifying predictors of assessment as ischemic heart disease (i.e. those with a univariate p<0.05 for association with IHD assessment), in all patients:

Age

Sex

Previous diabetes

Previous hypertension

Previous stroke

Previous chronic aspirin medication

Any chronic cardiovascular medication (other than aspirin)

Priority at the dispatch center

Pain/discomfort in chest

Pain/discomfort in neck

Pain/discomfort in arms

Pain/discomfort in stomach

Dyspnea symptoms

Cold sweat symptoms

Nausea symptoms

Paleness symptoms

Vertigo symptoms

Syncope symptoms

Heart rate >100 beats/min

Oxygen saturation <90%

ST-elevation on first in-hospital ECG

Pathologic T-wave on first in-hospital ECG

Left bundle branch block on first in-hospital ECG

Pathological Q-wave on first in-hospital ECG

Pulmonary rales on admission to hospital

Cardiogenic shock on admission to hospital

Baseline and in-hospital treatment variables used for adjustment when analyzing association between each of the six recommended pre-hospital medications and one-year mortality (i.e. those with an age-adjusted p<0.10 for association with mortality), in patients with no previous chronic aspirin medication:

Age

Sex

Previous myocardial infarction

Previous heart failure

Previous heart surgery

Previous stroke

Any chronic cardiovascular medication (other than aspirin)

Pain/discomfort in chest

Pain/discomfort in neck

Pain/discomfort in arms

Dyspnea symptoms

Paleness symptoms

Heart rate >100 beats/min

Oxygen saturation <90%

Assessed as ischemic heart disease by EMS

ECG recorded by EMS

Left bundle branch block on first in-hospital ECG

Pulmonary rales on admission to hospital

Cardiogenic shock on admission to hospital

Primary PCI

Anticoagulants in-hospital

Beta blockers iv in-hospital

Diuretics iv in-hospital

Inotropic drugs iv in-hospital

Nitroglycerine iv in-hospital

Coronary angiography in-hospital

Echo-cardiography in-hospital

Any PCI

**Table 1**

**Symptoms and initial assessment by dispatchers and EMS (%)**

_____________________________________________________________________________

Pre-hospital aspirin

All Yes No

(n=1726) (n=995) (n=731) p*

_____________________________________________________________________________

Priority at the dispatch center <0.0001#

1 78 84 68

2 21 15 29

3 1 <1 3

Priority by EMS <0.0001

1 66 86 40

2 30 14 51

3 4 <1 9

Pain/discomfort (10)** 93 >99 84 <0.0001

Localization of pain/discomfort## (13)**

Chest 95 96 93 0.004

Neck 14 15 11 0.02

Arms 41 46 32 <0.0001

Back 13 14 11

Stomach 5 3 8 0.0002

Other 2 1 2

Other symptoms (123)**

Dyspnea 30 23 39 <0.0001

Cold sweat 37 44 26 <0.0001

Anxiety 7 7 7

Nausea 24 27 20 0.001

Vomiting 10 11 9

Paleness 57 63 48 <0.0001

Vertigo 8 6 10 0.02

Syncope 5 3 6 0.003

Cardiogenic shock <1 <1 0

Other symptoms of heart failure <1 <1 <1

Hemodynamic

Systolic blood pressure <100mmHg(57)** 7 7 8

Heart rate >100 beats/min (57)** 19 14 27 <0.0001

Oxygen saturation <90% (102)** 11 6 18 <0.0001

Assessed as ischemic heart disease 84 96 67 <0.0001

ECG recorded 92 >99 82 <0.0001

_____________________________________________________________________________

* *age adjusted p-value, denoted if <0.05*

** *numbers within parenthesis: number of cases with missing information*

# *p-value refers to priority as an ordered variable*

## *of those with pain discomfort*

**Table 2.**

**Status on admission to hospital, treatment and investigation in hospital, 30 days and 1 year mortality (%)**

_____________________________________________________________________

Pre-hospital aspirin

All Yes No

(n=1726) (n=995) (n=731) p*

_____________________________________________________________________

STATUS ON ADMISSION

TO HOSPITAL

ST-T-pattern (16)**

ST-elevation 58 74 36 <0.0001

ST-depression 15 11 20 <0.0001

Pathologic T-wave 3 1 6 <0.0001

Other 10 7 14 0.0005

Normal 13 6 24 <0.0001

QRS-pattern (32)**

Pacemaker 1 <1 2

Left bundle branch block 7 6 9

Right bundle branch block 5 5 7

Pathological Q-wave 9 8 10

Other 6 4 9 0.0004

Normal 71 76 63 <0.0001

Heart failure

Pulmonary rales (103)** 10 5 15 <0.0001

Cardiogenic shock (48)** 2 2 3

TREATMENT AND

INVESTIGATION IN HOSPITAL

Reperfusion treatment (5)**

Primary PCI 58 75 36 <0.0001

Thrombolysis <1 <1 <1

CABG <1 <1 <1

Coronary angiography 4 4 5

without further treatment

None 36 20 58 <0.0001

Medication

Anticoagulants, iv/sc (4)** 44 32 61 <0.0001

Platelet inhibitors, iv (6)** 12 15 7 0.0003

Beta blockers, iv (4)** 5 4 5

Diuretics, iv (4)** 19 13 26 <0.0001

Inotropics, iv (4)** 4 3 5 0.02

Nitroglycerine, iv (2)** 12 9 16 <0.0001

Evaluation and treatment

Coronary angiography 85 93 74 <0.0001

PCI 74 86 57 <0.0001

Echo-cardiography (4)** 82 85 76 0.001

Left ventricular function# (51)**

*Cont.*

Normal 55 56 55

Slightly depressed 22 25 18

Moderate depressed 16 14 19

Severely depressed 6 6 8

MORTALITY

30 days 6.9 5.1 9.4

1 year 13.6 10.1 18.6 0.009

______________________________________________________________________

* *age adjusted p-value, denoted if <0.05*

** *numbers within parenthesis: number of cases with missing information*

# *of those where an echocardiography was performed*
